# A Hybrid Mean Value Involving Dedekind Sums and the General Exponential Sums

**DOI:** 10.1155/2014/914591

**Published:** 2014-08-07

**Authors:** Jianghua Li, Tingting Wang

**Affiliations:** ^1^College of Science, Xi'an University of Technology, Xi'an, Shaanxi 710048, China; ^2^College of Science, Northwest A&F University, Yangling District, Shaanxi 712100, China

## Abstract

The main purpose of this paper is using the analytic method, A. Weil's classical work for the upper bound estimate of the general exponential sums, and the properties of Gauss sums to study the hybrid mean value problem involving Dedekind sums and the general exponential sums and give a sharp asymptotic formula for it.

## 1. Introduction

For a positive integer *k* and an arbitrary integer *h*, the classical Dedekind sums *S*(*h*, *k*) are defined by

(1) $$\begin{array}{c} S\left(h,k\right)=\overset{k}{\underset{a=1}{\sum }}\left(\left(\frac{a}{k}\right)\right)\left(\left(\frac{ah}{k}\right)\right), \end{array}$$

where

(2) $$\begin{array}{c} \left(\left(x\right)\right)=\{\begin{array}{cc} x-\left[x\right]-\frac{1}{2}, & \text{if}\;x\;\text{is}\;\text{not}\;\text{an}\;\text{integer};\\ 0, & \text{if}\;x\;\text{is}\;\text{an}\;\text{integer}. \end{array} \end{array}$$

The various properties of *S*(*h*, *k*) were investigated by many authors; see [1–5]. For example, Conrey et al. [3] studied the mean value distribution of *S*(*h*, *k*) and proved the asymptotic formula

(3) ∑h=1k ′|S(h,k)|2m=fm(k)(k12)2m +O((k9/5+k2m−1+(1/(m+1)))·ln⁡3k),

where ∑_*h*_′ denotes the summation over all *h* such that (*k*, *h*) = 1, and

(4) $$\begin{array}{c} \overset{\infty }{\underset{m=1}{\sum }}\frac{f_{m}\left(n\right)}{n^{s}}=2\cdot \frac{\zeta^{2}\left(2m\right)}{\zeta \left(4m\right)}\cdot \frac{\zeta \left(s+4m-1\right)}{\zeta^{2}\left(s+2m\right)}\cdot \zeta \left(s\right). \end{array}$$

Zhang [6] established a close contact between *S*(*h*, *q*) and the mean square value of Dirichlet *L*-functions (see Lemma 4). Maybe the most important property of *S*(*h*, *q*) is its reciprocity theorem (see [2]). That is, for all positive integers *h* and *q* with (*h*, *q*) = 1, we have the identity

(5) $$\begin{array}{c} S\left(h,q\right)+S\left(q,h\right)=\frac{h^{2}+q^{2}+1}{12hq}-\frac{1}{4}. \end{array}$$

On the other hand, Liu and Zhang [5] studied the hybrid mean value problem involving *S*(*h*, *k*) and *R*_*q*_(*c*) and proved the following conclusion.

Let *q* > 2 be a square-full number; then

(6) ∑h=1q ′S(h,q)Rq(h+1)=−13·q·ϕ2(q)·∏p ∣ q(1+1p),

where *R*_*q*_(*c*) is the Ramanujan sum, defined as (see Theorem 8.6 of [7])

(7) $$\begin{array}{c} R_{q}\left(c\right)=\overset{q}{\underset{\overset{k=1}{\left(k,q\right)=1}}{\sum }}e^{2\pi ikc/q}=\underset{d\;|\;\left(c,q\right)}{\sum }d\mu \left(\frac{q}{d}\right), \end{array}$$

and *μ*(*n*) is the famous Möbius function.

Weil [8] studied the upper bound estimate of the general exponential sums

(8) $$\begin{array}{c} E\left(f\left(x\right),p\right)=\overset{p}{\underset{a=1}{\sum }}e\left(\frac{f\left(a\right)}{p}\right) \end{array}$$

and proved the estimate (see Corollary 2F of [9, page 45])

(9) $$\begin{array}{c} E\left(f\left(x\right),p\right)\ll_{k}\sqrt{p}, \end{array}$$

where *e*(*x*) = *e* ^2*πix*^, *p* is an odd prime, ≪_*k*_ denotes the big-*O* constant which depends only on *k*, and *f*(*x*) = *a*_*k*_ *x* ^*k*^ + *a*_*k*−1_ *x* ^*k*−1^ + ⋯+*a*_1_ *x* + *a*_0_ is *k*th integral coefficients polynomial with (*a*_*k*_, *p*) = 1.

In fact, the estimate (9) is the best one, since if *f*(*x*) = *x* ^2^, then from Gauss famous work (see [7, page 195]) we have

(10) $$\begin{array}{c} \left|\overset{p}{\underset{a=1}{\sum }}e\left(\frac{a^{2}}{p}\right)\right|=\sqrt{p}. \end{array}$$

The content and form of this paper are different from the references [3, 6]. Conrey et al. [3] studied the general 2*k*th power mean of Dedekind sums and obtained an asymptotic formula. Zhang [6] only obtained a relationship between *S*(*h*, *q*) and the mean square value of Dirichlet *L*-functions. Our work is using Zhang's result, Weil's classical work for the upper bound estimate of the general exponential sums, and the properties of Gauss sums to study the hybrid mean value problem involving Dedekind sums and the general exponential sums *E*(*f*(*x*), *p*) and give a sharper asymptotic formula for it.

## 2. Main Theorems

In this paper, we will obtain the following two results.

**Theorem 1 .**

Let *p* be an odd prime and let *k* be any fixed positive integer. Suppose that *f*(*x*) = *a*_*k*_ *x* ^*k*^ + *a*_*k*−1_ *x* ^*k*−1^ + ⋯+*a*_1_ *x* + *a*_0_ is a polynomial with integral coefficients having 0 < *k* < *p* and (*a*_1_, *p*) = 1. Then we have the asymptotic formula

(11) ∑m=1p−1∑n=1p−1E(mx+f(x),p)·E(nx−f(x),p)·S(m·n¯,p)  =112·p3+O(p5/2·ln⁡⁡p),

where $\bar{n}$ denotes the solution of the congruence equation *nx* ≡ 1mod⁡*p*.

For the classical Kloosterman sums, we can also obtain a similar conclusion. That is, we have the following.

**Theorem 2 .**

Let *p* be an odd prime; then we have the asymptotic formula

(12) ∑m=1p−1∑n=1p−1K(m+1,p)·K(n+1,p)·S(m·n¯,p)  =112·p3+O(p5/2·ln⁡p),

where $K(m,p)=\sum_{a=1}^{p-1}e\left(\left(ma+\bar{a}\right)/p\right)$ denotes the classical Kloosterman sums.

For general integer *q* > 3, whether there exists a similar asymptotic formula as in Theorem 1 (or Theorem 2) is an open problem.

## 3. Lemmas and Proofs of the Theorems

In order to complete the proof of our theorems, we need the following several simple lemmas. Hereinafter, we will use many definitions and properties of Gauss sums, Kloosterman sums, and character sums, all of which can be found in [7, 10–13], so they will not be repeated here. First we have the following.

**Lemma 3 .**

Let *p* be an odd prime and let *χ* be the Dirichlet character mod⁡*p*. Then one has the estimate

(13) $$\begin{array}{c} \overset{p-1}{\underset{r=1}{\sum }}\left|\underset{\overset{\chi \mathrm{mod}p}{\chi (-1)=-1}}{\sum }\chi \left(r\right){\left|L\left(1,\chi \right)\right|}^{2}\right|=O\left(p\cdot \ln p\right), \end{array}$$

where ∑_*χ*mod⁡*p*__*χ*(−1)=−1_ denotes the summation over all odd characters *χ*mod⁡*p*.

**Proof**

From the method of proving Lemma 5 in [14] we may immediately deduce this estimate.

**Lemma 4 .**

Let *q* > 2 be an integer; then for any integer *a* with (*a*, *q*) = 1, one has the identity

(14) $$\begin{array}{c} S\left(a,q\right)=\frac{1}{\pi^{2}q}\underset{d\;|\;q}{\sum }\frac{d^{2}}{\phi \left(d\right)}\underset{\overset{\chi \mathrm{mod}d}{\chi (-1)=-1}}{\sum }\chi \left(a\right){\left|L\left(1,\chi \right)\right|}^{2}, \end{array}$$

where *L*(1, *χ*) denotes the Dirichlet *L*-function corresponding to character *χ*mod⁡*d*.

**Proof**

See Lemma 2 of [6].

**Lemma 5 .**

Let *p* be an odd prime; then one has the asymptotic formula

(15) ∑χ(−1)=−1χmod⁡p|∑a=1p−1χ(a)·e(a+a¯p)|2·|L(1,χ)|2  =π212·p2+O(p3/2·ln⁡p).

**Proof**

See the theorem and corollary of [14].

**Proof of Theorem 1.**

From Lemma 4 with *q* = *p*, an odd prime, we have

(16) ∑χ(−1)=−1χmod⁡p|L(1,χ)|2=π2(p−1)p∑a=1p−1(ap−12)2=π212·(p−1)2(p−2)p2,

(17) ∑m=1p−1∑n=1p−1E(mx+f(x),p)·E(nx−f(x),p)·S(m·n¯,p)=pπ2(p−1)∑χ(−1)=−1χmod⁡p∑m=1p−1χ(m)E(mx+f(x),p)  ×∑n=1p−1χ¯(n)E(nx−f(x),p)|L(1,χ)|2=pπ2(p−1)∑χ(−1)=−1χmod⁡p|∑m=1p−1χ(m)E(f(x)+mx,p)|2 ·|L(1,χ)|2.

From the definition of *E*(*f*(*x*), *p*) and the properties of Gauss sum, we have

(18) ∑m=1p−1χ(m)E(f(x)+mx,p)  =∑a=1p−1∑m=1p−1χ(m)·e(f(a)+map)  =∑a=1p−1e(f(a)p)∑m=1p−1χ(m)·e(map)  =τ(χ)·∑a=1p−1χ¯(a)·e(f(a)p).

Note that for any nonprincipal character *χ*mod⁡*p*, we have $|\tau (\chi )|=\sqrt{p}$. For any integer 2 ≤ *a* ≤ *p* − 1, since (*a*_1_(*a* − 1), *p*) = 1, the polynomial *f*(*ax*) − *f*(*x*) satisfying *p*∤(*f*(*ax*) − *f*(*x*)). Applying (9), we have the estimate

(19) $$\begin{array}{c} \left|\overset{p-1}{\underset{b=1}{\sum }}e\left(\frac{f\left(ab\right)-f\left(b\right)}{p}\right)\right|\ll \sqrt{p}. \end{array}$$

Now combining (16), (17), (18), (19), and Lemma 3, we have the asymptotic formula

(20) ∑m=1p−1∑n=1p−1E(mx+f(x),p)·E(nx−f(x),p)·S(m·n¯,p)  =p2π2(p−1)∑χ(−1)=−1χmod⁡p|∑a=1p−1χ¯(a)·e(f(a)p)|2·|L(1,χ)|2  =p2π2(p−1)∑χ(−1)=−1χmod⁡p∑a=1p−1∑b=1p−1χ¯(a)χ(b)   ·e(f(a)−f(b)p)·|L(1,χ)|2  =p2π2(p−1)∑χ(−1)=−1χmod⁡p∑a=1p−1χ¯(a)∑b=1p−1e(f(ab)−f(b)p)   ·|L(1,χ)|2  =p2π2(p−1)∑a=2p−1(∑b=1p−1e(f(ab)−f(b)p))   ·(∑χ(−1)=−1χmod⁡pχ¯(a)|L(1,χ)|2)   +p2π2∑χ(−1)=−1χmod⁡p|L(1,χ)|2  =p2π2·π212·(p−1)2(p−2)p2   +O(p∑a=2p−1p·|∑χ(−1)=−1χmod⁡pχ¯(a)|L(1,χ)|2|)  =p2π2·π212·(p−1)2(p−2)p2   +O(p3/2·∑a=1p−1|∑χ(−1)=−1χmod⁡pχ¯(a)|L(1,χ)|2|)  =112·p3+O(p5/2·ln⁡p).

This proves Theorem 1.

**Proof of Theorem 2.**

Note that if *χ*(−1) = −1, then $\tau (\bar{\chi })=-\bar{\tau (\chi )}$. Consider

(21) ∑m=1p−1χ(m)K(m+1,p)  =∑a=1p−1∑m=1p−1χ(m)·e((m+1)a+a¯p)  =τ(χ)∑a=1p−1χ¯(a)·e(a+a¯p),∑a=1p−1χ¯(a)·e(a+a¯p)=χ¯(−1)·∑a=1p−1χ(a)·e(a+a¯p)¯.

From (16), the properties of Gauss sums, and Lemma 5, we have

(22) ∑m=1p−1∑n=1p−1K(m+1,p)·K(n+1,p)·S(m·n¯,p)=pπ2(p−1)∑χ(−1)=−1χmod⁡p∑m=1p−1χ(m)K(m+1,p) ·∑n=1p−1χ¯(n)K(m+1,p)|L(1,χ)|2=pπ2(p−1)∑χ(−1)=−1χmod⁡p|τ(χ)|2 ·|∑a=1p−1χ¯(a)·e(a+a¯p)|2·|L(1,χ)|2=p2π2(p−1)∑χ(−1)=−1χmod⁡p|∑a=1p−1χ(a)·e(a+a¯p)|2 ·|L(1,χ)|2=112·p3+O(p5/2·ln⁡p).

This completes the proof of Theorem 2.
